# Oxidation of Ethanol in the Brain and Its Consequences

**Published:** 2006

**Authors:** Richard Deitrich, Sergey Zimatkin, Sergey Pronko

**Affiliations:** Richard Deitrich, Ph.D., is a professor emeritus, Department of Pharmacology, University of Colorado, Aurora, Colorado. Sergey Zimatkin, M.D., Ph.D.,is a professor, Grodno State Medical University, Grodno, Belarus. Sergey Pronko, M.D., Ph.D., is a fellow, Department of Pharmacology, University of Colorado, Aurora, Colorado

**Keywords:** Ethanol metabolism, ethanol-to-acetaldehyde metabolism, acetaldehyde, acetate, aldehyde dehydrogenase (ALDH), central nervous system, brain, catalase, cytochrome P450, alcohol dehydrogenase (ADH), ethanol oxidation, behavior, ethanol preference

## Abstract

Acetaldehyde, a toxic byproduct of alcohol (i.e., ethanol) metabolism, has long been suspected of causing at least some of the central nervous system actions of ethanol. However, the data to support such a hypothesis have been difficult to obtain. One roadblock is the very low blood levels of acetaldehyde following ethanol intake and the finding that even elevated acetaldehyde levels in the blood do not easily gain access to the brain. The recent discovery of the oxidation of ethanol to acetaldehyde in the adult brain may help explain the acute effects of ethanol.

This article reviews studies of a potential role for acetaldehyde, a toxic byproduct of alcohol (i.e., ethanol) metabolism, in ethanol’s effects in the central nervous system (CNS); the metabolism of ethanol to acetaldehyde in the brain; the metabolism of acetaldehyde in brain cells; the results of ethanol oxidation to form acetalde-hyde; and acetaldehyde’s effects on behavior. The studies cited primarily are those dealing with acute or very short-term administration of ethanol. The role of acetaldehyde in tolerance and dependence or in the peripheral effects of ethanol is not covered.

## Acetaldehyde’s Role in Ethanol’s Effects

Acetaldehyde, a toxic byproduct of ethanol metabolism, may be at least partially responsible for ethanol’s actions in the CNS (Hunt 1996; Hashimoto et al. 1989; Bergamaschi et al. 1988; Zimatkin and Deitrich 1997; Thadani and Truitt 1977; Collins et al. 1988; Heap et al. 1995). However, several factors cast doubt on this hypothesis. First, avid metabolism of acetaldehyde by the liver keeps blood levels of acetaldehyde following ethanol ingestion extremely low (Sippel and Eriksson 1975). The levels of acetaldehyde in most people after ethanol ingestion are nearly undetectable in the blood, on the order of one micromole.^1^ Second, even if the blood acetaldehyde levels are significant, either because of genetic variation in alcohol-metabolizing enzymes or the presence of drugs that allow build-up of acetaldehyde, acetaldehyde does not seem to be able to penetrate blood vessels into the brain (i.e., the blood–brain barrier), and sub-stantial blood levels are required before acetaldehyde levels increase in the brain (Tabakoff et al. 1976; Westcott et al. 1980; Sippel 1974; Zimatkin and Pronko 1995). This is attributed primarily to the presence of the enzyme that converts acetaldehyde to acetate (i.e., aldehyde dehydrogenase [ALDH]) in the blood–brain barrier, which may help keep brain acetaldehyde levels low (Petersen 1985; Tampier et al. 1993). Third, although one could use the compound pyrazole to inhibit the reaction by which the enzyme alcohol dehydrogenase (ADH) breaks down ethanol (i.e., oxidation), and thus inhibit the formation of acetaldehyde, intoxication still would result, suggesting that acetaldehyde does not play a significant role in ethanol’s effects on the brain. Indeed, Goldstein and Zaechelein (1983) used pyrazole to study intoxication in mice using a vapor chamber method. In this method, the metabolism of inhaled ethanol is slowed, providing for more prolonged and consistent blood levels of ethanol, producing physical dependence in mice.

### Metabolism of Ethanol to Acetaldehyde in the Brain

These considerations would be irrelevant if the brain could produce its own acetaldehyde from ethanol. Although there had been several reports of the oxidation of ethanol in the brain (Sutherland et al. 1958; Raskin 1973; Raskin and Sokoloff 1974; Raskin and Sokoloff 1968; Raskin and Sokoloff 1970*a*, *b*; Raskin and Sokoloff 1972*a*, *b*), this idea largely was dismissed by the findings of Mukherji and colleagues (1975), whose studies showed that ethanol did not break down to acetaldehyde in the brain.

#### Catalase

Catalase, the enzyme that facilitates the breakdown of hydrogen peroxide to oxygen and water, may play a role in the production of acetaldehyde from ethanol in the brain. Cohen and colleagues (1980) demonstrated that catalase in conjunction with hydrogen peroxide will oxidize ethanol in the brain. Although the authors did not directly demonstrate the production of acetaldehyde (and thus the metabolism of ethanol), in this system, they did provide impetus to other investigators’ attempts. Researchers initially were thwarted in their attempts to document metabolism of ethanol in the brain when they discovered nonenzymatic (i.e., artifactual) production of acetaldehyde. It was determined that the iron typically found in red blood cells (i.e., hemoglobin) caused this nonenzymatic formation of acetaldehyde from ethanol, thus masking any enzymatic production of acetaldehyde. Two groups, nearly simultaneously, overcame these problems. Aragon and colleagues (1992) demonstrated that acetaldehyde was produced from ethanol in rat brains with all blood removed. Gill and colleagues (1992) were able to prevent the artifactual formation of acetaldehyde and show the production of acetaldehyde from ethanol in rat brain tissue. In both studies, inhibitors of catalase also were effective in inhibiting the production of acetaldehyde. On the other hand, inhibitors of the enzymes cytochrome P450 and ADH—key enzymes involved in alcohol metabolism— were ineffective. Other investigators quickly confirmed these findings (Hamby-Mason et al. 1997; Aspberg et al. 1993; Zimatkin et al. 1999). In studies of cells from all parts of the brain (i.e., whole-brain homogenates), the intensity of ethanol oxidation is comparatively low but may be much higher in the specific structures and cells known for their increased catalase activity (Zimatkin and Lindros 1996).

#### Cytochrome P450

Cytochrome P450 enzymes, which are involved in ethanol metabolism in the liver, have been implicated in the metabolism of ethanol in the brain. First, Warner and Gustafsson (1994) demonstrated the presence of cytochrome P450 in rat brain and its induction by ethanol. Cytochrome P450 2E1, a variant of cytochrome P450 (i.e., isozyme) that is capable of oxidizing ethanol efficiently in other tissues, also was found in the brain (Hansson et al. 1990). Its protein, messenger RNA (mRNA), specific activity, and induction by ethanol were found in nerve cells (i.e., neurons) and support cells (i.e., glial cells) in the following brain regions: cerebellum, cerebral cortex, thalamus, and hippocampus (Sohda et al. 1993; Tindberg and Ingelman-Sundberg 1996; Upadhya et al. 2000). Cytochrome P450 also has been found in prenatal human brain cells (Khalighi et al. 1999). In addition, a recent study in rats and mice has reinforced the possibility that cytochrome P450 is involved in the brain’s metabolism of ethanol (Zimatkin et al. 2006).

#### ADH

The possible role of ADH in the metabolism of ethanol in the brain remains unclear. Research originally suggested that only the subtype ADH3 was expressed in the brain and that ethanol was not a good candidate for that enzyme to act on (i.e., it was a poor substrate) (Beisswenger et al. 1985; Kerr et al. 1989). Several groups (Kerr et al. 1989; Buhler et al. 1983) reported the presence of ADH1 in brain cells. Giri and colleagues (1989) found ADH3 expressed in rat brain, and Martinez and colleagues (2001) found mRNA for ADH1 and ADH4 in rat brain cells. Although the authors could not demonstrate ADH activity in analysis of whole-brain homogenates, they were able to detect ADH activity in specific neurons (i.e., granular cells and Purkinje cells) of the cerebellum. This shows that although the activity of ADH may be undetectable in whole homogenates, there may be sufficient activity in specific neurons to form acetaldehyde locally.

## The Chemical Reactions Allowing Oxidation of Ethanol to Acetaldehyde

The oxidation of ethanol produces acetaldehyde (see Figure). The production of acetaldehyde by catalase is limited by the availability of hydrogen peroxide, a potentially harmful byproduct of ethanol metabolism by cytochrome P450. Hydrogen peroxide also can come from a number of other sources, including the enzyme monoamine oxidase, ascorbic acid (vitamin C), and other cytochrome P450 oxidations (Sandri et al. 1990; Simonson et al. 1993; Sinet et al. 1980).

All studies of the oxidation of ethanol to acetaldehyde depend on the ability to measure the accumulation of acetaldehyde. This can occur only if the rate of removal of acetaldehyde is slower than its rate of formation in the system under study. Substantial amounts of acetaldehyde are oxidized to acetate in these in vitro systems. This results in an underestimation of the rate of ethanol metabolism in the brain, because acetaldehyde is metabolized to acetate nearly as quickly as it is formed. Thus, only the net amount of acetaldehyde in the system is accounted for when only acetaldehyde accumulation is measured. Studies of the oxidation of ethanol to acetaldehyde in the brain therefore need to consider these limitations.

## Metabolism of Acetaldehyde in Brain Cells

The metabolism of acetaldehyde in the brain is much less controversial than the metabolism of ethanol because ALDH enzymes have long been known to be present in brain cells (Deitrich 1966; Erwin and Deitrich 1966). The ALDH enzyme most likely to be responsible for the majority of the oxidation of acetaldehyde to acetate is ALDH2, the form found in the mitochondria, an internal component of the cell. This enzyme has a high affinity (i.e., a low *K*_m_)^2^ and rate of enzyme activity with acetaldehyde and is sufficient to remove most of the acetaldehyde. Several other forms of ALDH are expressed in brain cells as well (Sophos and Vasiliou 2003). The localization of the enzyme to specific cells or areas of the brain could greatly influence the local rate of removal of acetaldehyde. That is, acetaldehyde is only metabolized if it is present in an area of the brain that also has ALDH enzymes (Zimatkin et al. 1992). In a similar fashion, localization within the cell of the enzymes responsible for the production of acetaldehyde (catalase in internal cell components called peroxisomes and cytochrome P450 in a network of membranes within the cell called the endoplasmic reticulum or microsomes) and the enzyme of acetaldehyde removal—ALDH2 in the mitochondria—leave space and time for acetaldehyde to interact with other cellular elements before being converted to acetate. That is, if acetaldehyde is produced in separate cellular structures from where it is removed, it can have an effect on the cell before it is metabolized.

Acetaldehyde’s conversion to acetate has further implications for the cell. Acetate has significant CNS effects that are separate from those of ethanol (Carmichael et al. 1991; Cullen and Carlen 1992; Correa et al. 2003). Thus, administering sodium acetate in doses comparable with those observed after administering 1 to 2 g/kg ethanol produced a dose-dependent impairment of motor coordination (Carmichael et al. 1991). Because acetate’s effects can be blocked with the use of 8-phenyltheophylline, a substance that blocks receptors for adenosine (a byproduct in acetate breakdown), it has been suggested that acetate’s actions may be mediated by adenosine (Carmichael et al. 1991; Cullen and Carlen 1992). Moreover, administering low doses of acetate (0.35 to 2.8 micromolar) by injection into the brain through a small hole bored into the skull (i.e., intracerebroventricular [ICV] administration) produced a potent decrease in motor activity, similar to the effects of ethanol and acetaldehyde on motor activity (Correa et al. 2003).

## Consequences of the Oxidation of Ethanol to Acetaldehyde

Ethanol oxidation to acetaldehyde has several consequences, which may be broken down into two broad categories. The first is the direct binding of acetaldehyde to proteins (Jennett et al. 1987; McKinnon et al. 1987; Nakamura et al. 2003; Zimatkin et al. 1992), nucleic acids (Wang et al. 2000), and a type of fat (i.e., lipid) containing phosphorus (i.e., phospholipids) (Trudell et al. 1990, 1991; Trudell et al. 1990; Kenney 1982, 1984). In total, the binding to these cellular components probably accounts for very little of the acetaldehyde that disappears, but the consequences of these interactions may be highly significant because the function of these cellular components can be compromised by this binding. The second category of ethanol oxidation consequences is indirect action. This occurs when the metabolism of other aldehydes that originate in the body (i.e., endogenous aldehydes) is inhibited through the presence of acetaldehyde. The aldehydes produced by the oxidation, by monoamine oxidase, of the brain chemicals (i.e., neurotransmitters) dopamine, norepinephrine, and serotonin, are particularly vulnerable to this reaction (Deitrich and Erwin 1980). One theory is that acetaldehyde or the aldehydes of dopamine, norepinephrine, or epinephrine condense with these same neurotransmitters to produce compounds called tetrahydroisoquinolines that may be responsible for some of ethanol’s CNS effects themselves. Acetaldehyde also may condense with serotonin to form compounds called tetrahydro-beta carbolines that may be active in the brain as well. Thus, these amine-aldehyde condensation products may be responsible for some of the behavioral actions of ethanol (Davis and Walsh 1970; Cohen and Collins 1970; Melchior and Myers 1977). Considerable controversy arose around this theory, especially because these compounds occur naturally in the diet, casting doubt on the relevance of their presence following ethanol ingestion (Collins et al. 1990).

Researchers recently have proposed that acetaldehyde may compete with malondialdehyde or 4-hydroxynonenal, the aldehyde products that result after the breakdown of lipids (i.e., lipid per-oxidation). These aldehydes also may inhibit the activity of ALDHs (Luckey et al. 1999; Mark et al. 1997; Meyer et al. 2004; Murphy et al. 2003). This would result in increased levels of acetaldehyde as well as of these toxic aldehydes.

The oxidation of ethanol to acetaldehyde may trigger various reactions that ultimately have behavior-related consequences, as described below.

## Acetaldehyde and Behavior

Several studies have suggested that acetaldehyde is responsible for some of the behavioral effects (such as poor coordination [i.e., ataxia]) of ethanol (reviewed in Deitrich 2004 and Quertemont et al. 2005). Many of these studies measured the degree of relationship of the two variables (i.e., they were correlational). That is, the studies measured the behavioral effects of ethanol following presumed alteration of levels of acetaldehyde in the brain by inhibiting ALDH or inhibiting or activating catalase.

Acetaldehyde has been measured directly in the brain in only a few studies (Jamal et al. 2003, 2004, 2005). In those studies, ALDH was inhibited, resulting in relatively high levels of acetaldehyde in the brain. It is assumed that the proximal cause of these behavioral effects (such as ataxia or loss of the righting response) is the altered amount of acetaldehyde in the brains of the animals studied.

On the other hand, mice with about half the usual levels of catalase in the brain (i.e., acatalasemic mice), which should have less acetaldehyde from ethanol in the brain compared with control mice, had longer sleep times after ethanol intake than control mice (Aragon and Amit 1993; Vasiliou et al. 2004). This suggests that acetaldehyde does not influence ethanol-induced sleep times. Similar results were obtained using mice that had been genetically modified to have the ethanol-metabolizing enzyme CYP2E1 absent. The mice exhibited longer ethanol-induced sleep times, especially at higher ethanol doses; they also produced lower amounts of acetaldehyde following the incubation of ethanol with brain cell structures containing ethanol-metabolizing enzymes (i.e., microsomes) compared with control animals (Vasiliou et al 2004). In addition, induction of brain catalase activity resulted in decreased loss of righting reflex (LORR), which is used to estimate hypnotic sensitivity (a behavioral response) to ethanol, whereas reduced brain catalase activity resulted in increased LORR, showing involvement of brain catalase in the hypnotic effect of ethanol (Correa et al. 2001).

### Ethanol Preference

Rats selectively bred to have either high or low preference for ethanol are useful animal models for the study of alcohol consumption. Alcohol-preferring (P) and nonpreferring (NP) rats have been shown to differ in hypnotic sensitivity to ethanol; thus, P rats are innately less sensitive to the effects of ethanol than NP rats (Lumeng et al. 1982).

Researchers have used P and NP pairs of rat lines or strains to study relationships between catalase, acetaldehyde, and ethanol preference. Although none of the studies below included direct measurements of brain ethanol oxidation in vitro, brain acetaldehyde levels in vivo, or acetaldehyde accumulation in vitro, their findings do offer some implications regarding the relationship between brain acetaldehyde and ethanol preference.

As discussed previously, catalase plays a role in the production of acetaldehyde in the brain. The catalase inhibitor aminotriazole attenuated ethanol preference in mice (Koechling and Amit 1994), suggesting that inhibiting catalase results in decreased levels of acetaldehyde and a decline in this particular ethanol-induced behavior. Consistent with these findings, Amit and Aragon (1988) found that blood catalase from rats naïve to ethanol correlated positively with ethanol preference in the animals (blood and brain catalase also correlated positively after exposure to ethanol). Increased catalase presumably would mean an increased rate of production and increased brain levels of acetaldehyde with resultant increases in preference. Similar studies have found that catalase correlates with alcohol intake in humans as well (Koechling and Amit 1992).

ALDH in the brain also positively correlates with ethanol preference. Indeed, Amir (1978) found that rats’ ethanol preference better correlated with brain ALDH than with liver ALDH. This would support the idea that blood levels of acetaldehyde (produced in the liver) are less important to establishing a preference for alcohol than brain levels of acetaldehyde. High blood acetaldehyde levels, resulting from a genetic defect in ALDH in Asian populations, do produce decreased ethanol intake (Harada et al. 1982), as does treatment with ALDH inhibitors such as disulfiram (Antabuse^®^) (Chick et al. 1992). High ALDH activity would presumably indicate a lower level of brain acetaldehyde because of increased rates of oxidation. Higher ALDH and lower acetaldehyde levels are not consistent with the positive correlation between catalase activity and ethanol preference. Also, acatalasemic mice have a higher preference for ethanol than do control mice (Aragon and Amit 1993). Vasiliou and colleagues (2006) reported that acatalasemic mice accumulate only about 50 percent as much acetaldehyde from ethanol in vitro as control mice and that they have about half the brain catalase activity as that in the brain of control mice. This would not totally explain the decreased ethanol metabolism rates because catalase is not the only enzyme capable of oxidizing ethanol in the brain. Previous studies on catalase contribution to ethanol oxidation were performed using catalase inhibitors capable of inhibiting the activity of other ethanol-metabolizing enzymes (P4502E1 and ALDH); therefore, this finding using genetics seems to be a more useful tool. Other researchers have conducted extensive research that provides further support for the involvement of brain catalase in ethanol-induced behavioral effects. This research also supports the notion that acetaldehyde may be produced directly in the brain by catalase and that it may be an important regulator of ethanol’s locomotor effects (for example, see Sanchis-Segura et al. 1999).

### Acetaldehyde’s Actions in the Brain

Researchers also have studied the actions of acetaldehyde in the brain directly, usually with ICV infusions of acetaldehyde to bypass the metabolism of acetaldehyde by the liver. Smith and colleagues (1984) found that conditioned place preference^3^ could be induced by ICV infusions of acetaldehyde, suggesting that low levels of acetaldehyde have reinforcing properties. Brown and colleagues (1979) had found that rats would administer acetaldehyde, but not ethanol, intracerebroventricularly. Conversely, conditioned taste aversion can be induced by acetaldehyde. This action can be blocked by alpha-methyl-para-tyrosine, a substance that inhibits the neurotransmitters epinephrine (adrenaline) and dopamine—key brain chemicals involved in addiction. This indicated that perhaps the adrenergic system is involved in acetaldehyde’s action in the brain (Aragon et al. 1991). Rodd-Henricks and colleagues (2000, 2002) found that rats genetically predisposed to prefer alcohol would press a lever to infuse ethanol and acetaldehyde directly into the ventral tegmental area (VTA), located in the midbrain. Using similar techniques, it was found that rats would lever press for infusions of the condensation product between acetaldehyde and dopamine (i.e., salsolinol) directly into the nucleus accumbens, a collection of neurons involved in the brain’s reward system (McBride et al. 2002). Arizzi and colleagues (2003) studied the in vivo effects of intracerebroventricularly administered ethanol, acetaldehyde, and acetate on lever-pressing tasks. These studies showed that acetaldehyde appears to induce activating or disinhibiting effects and thus can produce at least some of the effects of ethanol, whereas acetate is more potent than the other substances at producing actions that lead to a suppression of lever pressing and locomotion and thus may be implicated in the motor impairments induced by ethanol. Unfortunately, the researchers gave the same dose of all three agents in spite of the large (1,000-fold) difference in their concentrations following ethanol administration peripherally.

Numerous studies show the possible pathophysiological effects of acetaldehyde. The theories of the condensation of acetaldehyde with biogenic amines to produce new compounds are reviewed above. Although this reaction certainly occurs in vivo, the importance of these condensation products to ethanol’s actions in the brain remains speculative. In a similar vein, acetaldehyde, by substrate competitive inhibition of ALDH, has been postulated to cause an increase in the aldehydes derived from biogenic amines (Deitrich and Erwin 1980). That is, ALDH can oxidize acetaldehyde and aldehydes derived from biogenic amines. When acetaldehyde is absent ALDH can oxidize other aldehydes. When acetaldehyde is present, acetaldehyde is bound to ALDH and is oxidized so other aldehydes are oxidized at a slower rate or not at all by ALDH. No studies have directly measured the purported increase in these aldehydes. However, these aldehydes do have suggested physiological actions. For example, indole-3acetaldehyde, a biogenic aldehyde, reacts with certain substances (i.e., phospholipids) to create specific physiological effects, as indicated by a change in spectrophotometric absorption^4^ (Nilsson and Tottmar 1985). This shows what can happen to biogenic aldehydes if they are not oxidized by ALDH. When aldehydes were directly applied to neurons, the biogenic aldehydes derived from dopamine and serotonin had a direct depressant effect on neurons in the neocortex and cerebellum (Palmer et al. 1986).

Many other studies of the direct actions of acetaldehyde are available. For example, large doses of acetaldehyde given ICV caused decreases of dopamine, serotonin, and a product of dopamine metabolism (i.e., a metabolite) (i.e., homovanillic acid [HVA]) and increases in a metabolite of serotonin (i.e., 5-hydroxyindoleacetic acid [5HIAA]) in an analysis of fluid from the nucleus accumbens (Ward et al. 1997). These and similar reports do not provide a consistent dataset from which likely mechanisms can be deduced. Part of the problem is that accurate measurements of acetaldehyde in the brain tissue have been difficult (see Westcott et al. 1980), and so no systematic correlation of acetaldehyde brain levels with behavioral effects has been carried out. Mascia and colleagues (2001) studied the effect of acetaldehyde on cloned neurotransmitter receptors in frog oocytes. Of those studied, only a receptor for the amino acid glycine was sensitive to acetaldehyde.

In summary, research has now provided ample evidence that ethanol is metabolized to acetaldehyde and then acetate in the brain. Several studies also suggest that the presence of acetaldehyde in the brain is responsible for at least some of the effects of ethanol.

The field will advance most rapidly by simultaneous measurement of acetaldehyde in the brain or brain areas and correlating these levels with specific behavioral actions.

## Figures and Tables

**Figure:**
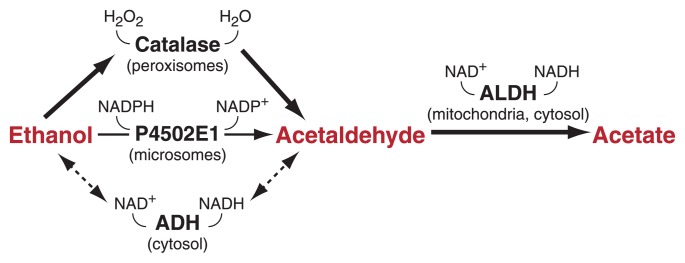
Pathways of ethanol metabolism in the brain. The oxidation of ethanol produces acetaldehyde. The production of acetaldehyde by the enzyme catalase (found in internal cell components called peroxisomes) requires hydrogen peroxide (H_2_O_2_). The enzyme cytochrome P4502E1 is present in brain cell structures in the smooth endoplasmic reticulum (microsomes). Alcohol dehydrogenase (ADH) is an enzyme found in the cell’s fluid or cytosol. The enzyme aldehyde dehydrogenase (ALDH), found in the cell’s mitochondria and cytosol, converts acetaldehyde to acetate.

## Footnotes

1 A micromole represents a concentration of 1/1,000,000 (one millionth) molecular weight per liter (mol/L).

2 *K*_m_ is a measurement used to describe the activity of an enzyme. It describes the concentration of the substance upon which an enzyme acts at which the enzyme works at 50 percent capacity.

3 To induce conditioned place preference, an animal is injected with the drug being studied and is placed in a test chamber with distinctive environmental cues. This procedure is repeated for several days. During these conditioning trials, the animal develops an association between the subjective state produced by the drug and the environmental cues present during the drug state. When the subject is tested in an apparatus that contains the drug-related environmental cues in one compartment and neutral cues in another, it voluntarily moves toward the compartment containing the drug-related cues.

4 Spectrophotometry is a determination of the concentration of a material in a sample by measurement of the amount of light the sample absorbs.
